# Characterization of aerosols generated during suspected aerosol-generating procedures in healthcare settings

**DOI:** 10.1017/ash.2026.10413

**Published:** 2026-05-21

**Authors:** Caroline A. O’Neil, Jiayu Li, Meghan A. Wallace, Ramesh Raliya, Yang Wang, Anna Leavey, Traci L. Bricker, Carey-Ann D. Burnham, Adrianus C.M. Boon, Pratim Biswas, Hilary M. Babcock

**Affiliations:** 1 Washington University In St Louis: Washington University in St Louis, USA; 2 University of Miami College of Engineering, USA; 3 Infectious Disease, Washington University School of Medicine, St Louis, USA

## Abstract

**Objective:**

To measure aerosol generation during various medical procedures that are potentially aerosol generating and determine whether these aerosols contain viral and/or bacterial pathogens that may pose a risk to healthcare personnel.

**Design:**

Observational.

**Setting:**

Tertiary care academic hospital.

**Patients:**

Convenience sample of adult patients undergoing one of the following procedures: extubation, bronchoscopy, mechanical ventilation, noninvasive ventilation, suctioning, nebulized medication administration (NMA), sputum induction, nasopharyngeal swab, or tracheostomy change.

**Methods:**

Four aerosol characterization instruments measured aerosol characteristics (particle mass, number, size); average baseline and procedure measurements were compared to identify changes in aerosolized particles associated with each type of procedure. SKC BioSamplers were used for viral and bacterial pathogen recovery. Clinical data were reviewed to examine patient characteristics that might impact pathogen recovery.

**Results:**

Among 93 sampled procedures, differences between baseline versus procedure measurements were only notable for NMA and sputum induction. Smaller increases in some particle measurements were observed for the single extubation sample. None of the 248 BioSampler specimens were positive for a respiratory virus, even though for 39 procedures, the patient had a recent clinical specimen that was positive for a respiratory virus. Thirty-two samples (13%) had positive bacterial cultures, all of which represented common skin/environmental contaminants or upper respiratory microbiota.

**Conclusions:**

In this study, significant aerosol generation was only observed during NMA and sputum induction. No viral pathogens and minimal bacteria were recovered from these medically generated aerosols. These data suggest that some procedures that are considered “aerosol-generating” may pose little infectious risk to healthcare personnel.

## Introduction

Most human pathogens spread from person to person through direct contact or through the air, via a range of particle sizes. The 2007 HICPAC guidelines recommend isolation precautions for patients with certain conditions, including “droplet precautions” for pathogens understood to spread through the air by larger particles (5–10 µm).^1^ Although use of a respirator has not been routinely recommended to protect healthcare personnel (HCP) from exposure to these pathogens,^1^ during the 2003 SARS outbreak, a series of case reports of infections among HCP raised widespread concerns that some pathogens, which usually spread by direct contact or larger airborne particles, could also be transmitted by smaller aerosolized particles (<5 µm) that may be generated during certain medical procedures.^2–5^ Concerns about aerosol-generating procedures re-arose during the 2009 H1N1 Influenza pandemic^6–8^ and again during the COVID-19 pandemic,^9–12^ which led to discussion about the epidemiologic definition of an aerosol, which is based on disease transmission, versus the aerosol science definition for an aerosol, which is based on particle size.^13^ Given concerns about aerosol generation and disease transmission, some infection prevention guidelines therefore recommend that HCP use additional respiratory protection (eg, fitted particulate respirators) when performing “aerosol-generating procedures.”^1,14–16^

Despite these concerns, questions remain about which procedures are associated with increased risk, the extent to which small particle aerosols are generated during these procedures, the size and concentration of medically aerosolized particles, and whether such aerosols can transmit viable pathogens to HCP or other patients.^17–19^ Routine medical procedures often considered to be “aerosol-generating” include: endotracheal intubation and extubation, cardiopulmonary resuscitation, bronchoscopy, non-invasive ventilation, tracheotomy sputum induction, airway suctioning, manual ventilation, and administering high-flow oxygen or nebulized medication.^1,17,18,20–23^ For most of these procedures, evidence for the generation of infectious aerosols is based mainly on case reports and retrospective investigations rather than on epidemiological studies or environmental air sampling. Further, multiple reviews have concluded that, for several types of procedures that are considered aerosol-generating, there is insufficient evidence to confirm they actually produce aerosols or are associated with increased risk of viral transmission.^11,20,23^

Environmental sampling during suspected aerosol-generating procedures has commonly focused on pathogen recovery from the air in clinical spaces rather than on the characterization of aerosolized particles.^24,25^ Methods used for pathogen recovery are varied and most have significant limitations.^26^ Many studies rely on molecular detection (eg, PCR) which can indicate the presence of a pathogen’s genetic material but cannot determine viability or ability to cause infection.^24,27,28^ Studies that include testing to determine pathogen viability, such as viral culture, are less common.^29,30^

A pilot study conducted at our institution examined aerosol generation during 6 routine healthcare procedures and tested samples for bacterial pathogens.^31^ However, only a limited number of procedures were evaluated, and we did not test for the presence of viruses or evaluate patient-level factors. The aim of the current study was to characterize the aerosols generated during an expanded list of potentially aerosol-generating procedures and to determine the presence and viability of viral and bacterial pathogens in medically generated aerosols to evaluate the disease transmission risk to HCP.

## Methods

Nine potentially aerosol-generating procedures were targeted for sampling: extubation, bronchoscopy, mechanical ventilation, noninvasive ventilation (bilevel positive airway pressure or continuous positive airway pressure), suctioning (open airway and/or tracheostomy), nebulized medication administration (NMA), sputum induction, collection of nasopharyngeal (NP) swabs, and tracheostomy change. Samples were collected between January 19, 2017 and June 27, 2018 in the medical intensive care unit (MICU), tracheostomy care unit, outpatient bronchoscopy suite, and outpatient respiratory therapy procedure room at a 1,272-bed tertiary care academic hospital. Patients age ≥18 years who underwent any of the targeted procedures were eligible for aerosol sampling, with prioritization of patients who had a clinical test positive for a respiratory virus within the previous 7 days. The study protocol was reviewed and approved by the Washington University Human Research Protection Office. Verbal assent was obtained from patients and healthcare workers prior to sampling.

### Aerosol sampling

Four real-time aerosol characterization instruments were used to measure aerosols generated during the targeted procedures. The P-Trak Ultrafine Particle Counter (TSI, Inc.) measured particle number concentration (number/cm^3^); the SidePak AM520 Personal Aerosol Mobility Spectrometer (TSI, Inc.) measured particle mass concentration (PM_2.5_; mg/cm^3^); the AeroTrak Portable Particle Counter (TSI, Inc.) estimated particle surface area (µm^2^/cm^3^) that would deposit in the alveolar region of the human lung; and the Aerodynamic Particle Sizer measured the size distribution of aerosolized particles ranging from 0.5–20 µm. The instruments were mounted on a mobile sampling cart and positioned approximately 3 feet from the patient during sampling. See methods supplement for detailed descriptions of the instruments.

For most procedures, baseline measurements were collected before the procedure, whereas the patient was in the room. When this was not possible (as in the case of emergent procedures), baseline measurements were collected ≥ 15 minutes after the procedure. No baseline measurements were collected for continuous procedures (mechanical ventilation and noninvasive ventilation), so the overall average of baseline measurements from other procedures was used for comparison.

For each procedure, the baseline and procedure measurements for particle mass, number, and surface area were separately averaged. The difference between the average baseline and procedure measurements was calculated to determine whether there was an increase in aerosolized particles during the individual procedure. At the end of the study period, baseline and procedure measurements from all samples were summarized, and for each procedure type, the maximum, minimum, and median percent change were calculated, and boxplots were generated. Average baseline versus procedure measurements of particle size distribution were compared graphically.

### Pathogen recovery

Sterile 5 ml SKC BioSamplers filled with 4 ml phosphate-buffered saline were used to collect specimens for pathogen recovery. Prior to 11/17/2017, two BioSamplers were used during each baseline and procedure sample: one was placed approximately 3 feet from the patient to estimate HCP exposure while the other was placed approximately 6 feet away to estimate the exposure of others in the room. Preliminary analysis of microbiology data showed no difference between the 3 ft and 6 ft specimens, so only the 3 ft BioSampler was used after 11/17/2017. The BioSamplers operated for the duration of each procedure. For bronchoscopies, one sampler was used during initiation and the first 15 min of the procedure and a second sampler was used during the last 10–15 min.

After sampling, all baseline and procedure specimens underwent analysis for detection of bacteria, including: Gram-stain; plating on blood agar, chocolate agar, and MacConkey agar; and broth enrichment (TSB with 6.5% NaCl), all incubated at 35° C. If the patient undergoing the procedure had a clinical specimen positive for any respiratory virus within 7 days of aerosol sampling, specimens were also tested using the Biofire FilmArray Respiratory Panel. If the patient had a clinical or study sample positive for influenza, the specimens also underwent testing with the Cepheid Xpert FLU/RSV assay, in addition to viral culture to identify the presence of viable influenza virus.

### Clinical data collection

The electronic medical record for each patient was reviewed to collect patient-level factors that might affect aerosol transmission dynamics or pathogen recovery, including age; respiratory symptoms; medications (antivirals, antibiotics, and steroids); and certain comorbidities, including COPD, heart disease, diabetes, kidney disease, and cancer. Microbiology reports from the date of hospital admission until 7 days after sampling were reviewed to identify clinical specimens that were positive for a respiratory virus. Details about the patient’s hospital admission were also collected, including inpatient/outpatient status, date of admission, and whether the patient was on droplet or contact precautions at the time of sampling. Study data were managed using REDCap electronic data capture tools hosted at Washington University.^32^

## Results

A total of 93 procedures were sampled: 1 extubation, 17 bronchoscopies, 20 mechanical ventilation, 17 noninvasive ventilation, 13 suctioning, 17 NMA, 6 sputum induction, 1 NP swab collection, and 1 tracheostomy change. In 38 of the 93 procedure samples (41%), the patient undergoing the procedure had a clinical specimen positive for a respiratory virus within 7 days of aerosol sampling. Sixteen patients were positive for influenza, and 21 were positive for one or more other respiratory viruses, including rhinovirus (9), parainfluenza (6), seasonal coronavirus (5), respiratory syncytial virus (3) and adenovirus (1). One patient was positive for both influenza and coronavirus.

Most aerosol samples were collected in the MICU (69 samples, 74%). However, 13 bronchoscopy samples were captured in the outpatient bronchoscopy suite (14%), the 6 sputum induction and 3 NMA samples were captured in the outpatient respiratory therapy procedure room (10%), and 2 suctioning samples were collected in the tracheostomy care unit (2%).

### Aerosol characterization data

**Figure 1.**
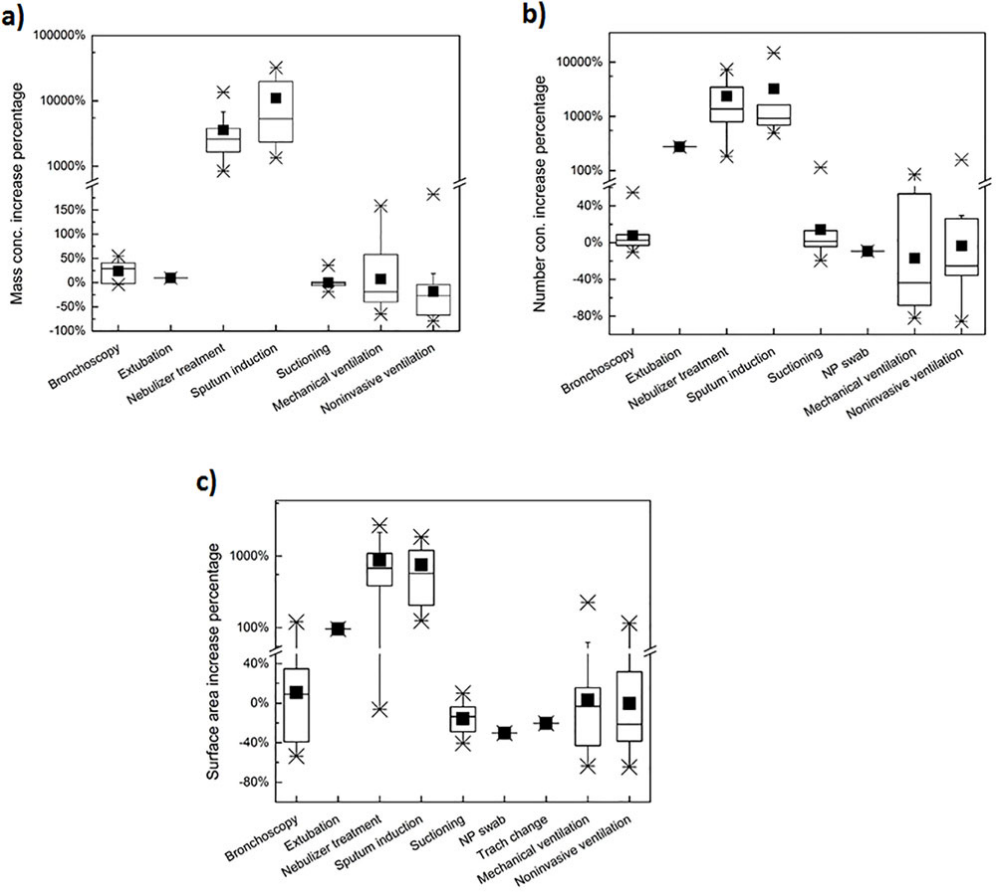
Percent increase in average procedure versus average baseline measurements of particle mass concentration (PM_2.5_) (a), particle number concentration (b), and particle surface area deposition, alveolar range, (c) by type of procedure. For continuous procedures (mechanical ventilation and noninvasive ventilation), average procedure measurements were compared to an overall average of baseline measurements from the other procedures. Key for the boxplots: top x = maximum value, top line = 95^th^ percentile, top of box = 75^th^ percentile, middle line in box = median, black square = mean; bottom of box = 25^th^ percentile, bottom line = 5^th^ percentile, and bottom x = minimum value. Only surface area measurements were available for the tracheostomy change procedure. Particle mass data was not available for the NP swab procedure. Note: both linear and logarithmic scales were used to better show the whole span of data.

The percent change in average procedure versus baseline measurements of particle mass concentration, particle number concentration, and particle surface area deposition for each type of procedure are shown in Figure 1. Baseline and procedure measurements of particle mass concentration were similar for all procedures except NMA and sputum induction, which uses nebulized saline to promote coughing (Figure 1a). Procedure measurements of particle number concentration (Figure 1b) and particle surface area deposition (Figure 1c) were also higher than baseline measurements for NMA and sputum induction. A smaller increase in particle number concentration and particle surface area deposition was observed during the single extubation procedure (Figure 1b and c). No notable differences in procedure versus baseline particle measurements were observed for mechanical ventilation, noninvasive ventilation, suctioning, bronchoscopy, or the single NP swab collection or tracheostomy change. Although the median percent increase in average procedure versus baseline measurements of particle mass concentration was slightly elevated for bronchoscopy samples (30% increase), this was much lower than the median percent increase for NMA or sputum induction, both of which exceeded 1,000% (Figure 1a).

**Figure 2.**
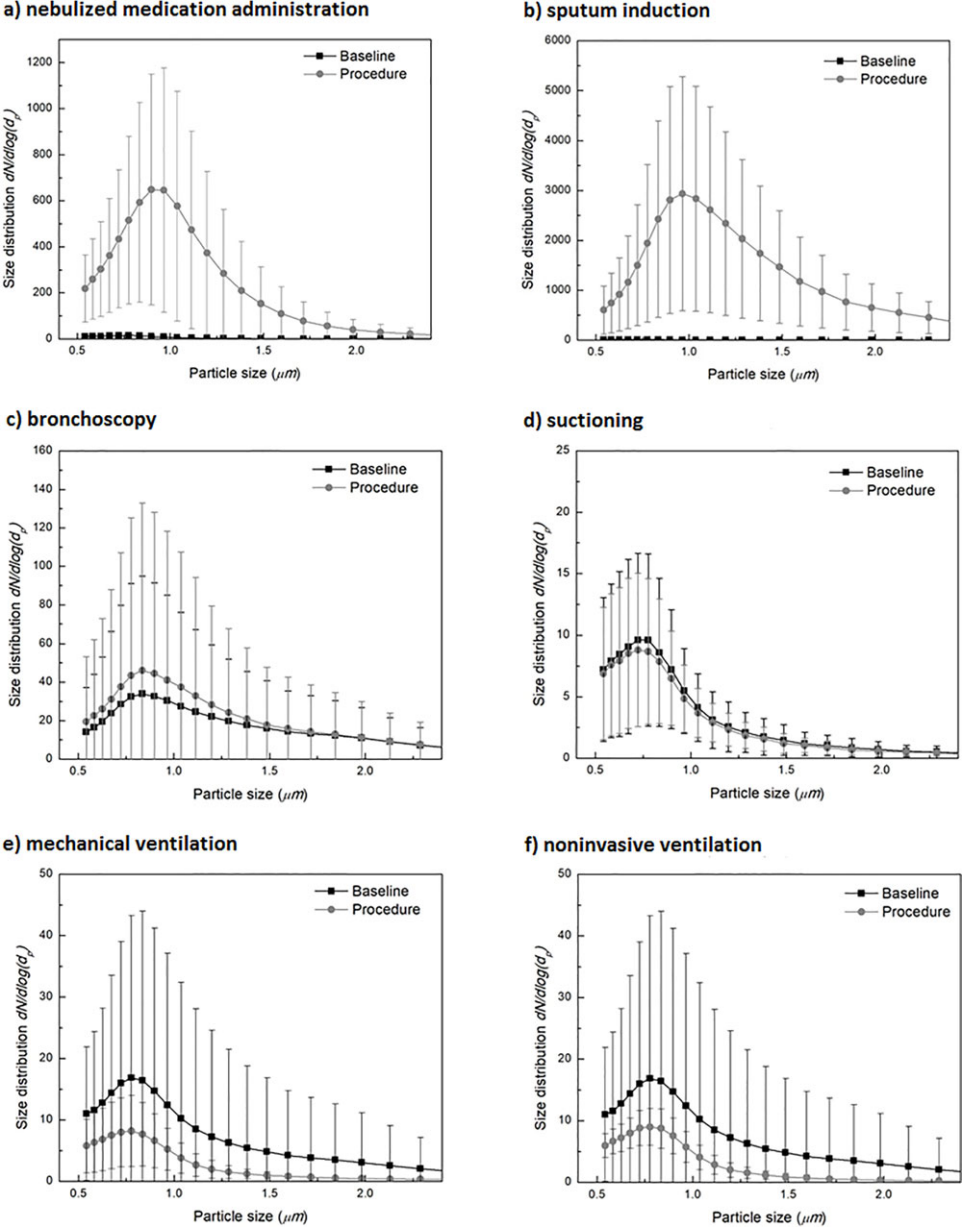
Average size distribution of detectable particles for baseline and procedure samples by type of procedure. For continuous procedures (mechanical ventilation and noninvasive ventilation), average procedure measurements were compared to an overall average of baseline measurements from the other procedures. The Y-axis shows particle quantity, while the X-axis shows particle size in microns. Note: scale of the Y-axis varies; data have been cropped at 2.5 µm to better show the peak.

Averaged baseline and procedure particle size distribution measurements for each procedure are shown in Figure 2. These data were not available for extubation, NP swab collection, or tracheostomy change due to equipment malfunction. Increases in particle quantity were observed during NMA (Figure 2a) and sputum induction (Figure 2b), but there was no difference in the concentration of particles detected for bronchoscopy (Figure 2c) and suctioning (Figure 2d) compared to baseline. There was no shift in the size distribution of detectable particles during procedure versus baseline sample periods for any procedure. For mechanical ventilation (Figure 2e) and noninvasive ventilation (Figure 2f), the concentration of particles was small, and particle size distribution curves were similar to the average baseline curve for the other procedures, suggesting no change in the sizes of detectable particles.

### Pathogen recovery data

A total of 248 SKC BioSampler specimens were submitted for pathogen recovery. No specimen tested positive for any respiratory virus using nucleic acid amplification methods, including 39 specimens collected during procedures performed on patients who had a clinical sample positive for a respiratory virus within 7 days of aerosol sampling. None of the specimens from the 17 procedures performed on patients who had a clinical specimen positive for influenza had a positive viral culture.

Thirty-two specimens (13%) had positive bacterial cultures; in seven, two types of bacteria were identified (Supplement Table 1). These bacteria represent common skin or environmental contaminants and upper respiratory microbiota. No drug-resistant bacteria were identified, including in specimens collected during the 35 procedures (38%) performed on patients who were on contact precautions for MRSA, VRE, or other MDROs at the time of aerosol sampling.

### Chart review data

The 93 procedures sampled for this study were performed on 82 patients. Most (73%) were hospital inpatients, with the average time between admission and aerosol sampling being 6.3 days (range 0–38 d). Demographic characteristics and comorbidities of the sampled patients are summarized in Supplement Table 2. Most patients (95%) had at least one significant underlying health condition, half (50%) were on droplet precautions for a respiratory virus at the time of sampling, and 31 (38%) were on contact precautions for drug-resistant bacteria. Thirty-three patients (40%) had a clinical specimen positive for a respiratory virus within 7 days of aerosol sampling. Comparisons of the respiratory symptoms reported by patients with versus without positive clinical specimens, and for patients with clinical specimens positive for influenza versus another respiratory virus are provided in Table 1. The mean number of days between earliest symptom onset and date of sampling (excluding patients with chronic respiratory symptoms lasting > 30 d) was 9.3 (range 0–28 d).

**Table 1.** Respiratory symptoms reported by and medications taken by patients with versus without a clinical sample positive for influenza or another respiratory virus in the 7 days before/after aerosol sampling

	All Patients *N* = 82 (%)	Patients with a clinical sample positive for RV^a^ *N* = 33 (%)	Patients without a clinical sample positive for RV *N* = 49 (%)
Symptoms reported			
Shortness of breath	51 (62.2)	23 (72.7)	27 (55.1)
Fever (≥ 99.1 °F)	50 (61.0)	23 (69.7)	27 (55.1)
Cough	39 (47.6)	18 (54.5)	21 (42.9)
Myalgia	4 (4.9)	2 (6.1)	2 (4.1)
Congestion or runny nose	11 (13.4)	5 (15.2)	6 (12.2)
Sore throat	3 (3.7)	1 (3.0)	2 (4.1)
Headache	5 (6.1)	1 (3.0)	4 (8.2)
Sputum production	28 (34.1)	12 (36.4)	16 (32.7)
Any symptom	72 (87.8)	31 (93.9)	41 (83.7)
Medications taken			
Antiviral	33 (40.2)	22 (66.7)	11 (22.4)
Antibiotic	68 (82.9)	30 (90.9)	38 (77.6)
Antifungal	26 (31.7)	9 (27.3)	17 (34.7)
Steroids (PO or IV)	45 (54.9)	21 (63.6)	24 (49.0)
Immunosuppressive	19 (23.2)	4 (12.1)	15 (30.6)

RV, respiratory virus.

a Fourteen patients were positive for influenza, eight were positive for rhinovirus, four were positive for parainfluenza, three were positive for seasonal coronavirus, two were positive for respiratory syncytial virus, one was positive for both influenza and seasonal coronavirus, and one was positive for rhinovirus, parainfluenza, and adenovirus.

Patient medication data are shown in Table 1. A higher proportion of patients with versus without a respiratory virus had received an antiviral medication (67% vs 22%), antibiotic (91% vs 78%), or a systemic (oral or intravenous) steroid (64% vs 49%) in the 7 days before aerosol sampling. Of the 15 patients who had a clinical specimen positive for influenza in the 7 days before sampling, 14 had received Oseltamivir (Tamiflu) for a mean of 2 days (SD, 2.6; median, 1.0; range, 0–9) prior to the date of aerosol sampling.

## Discussion

In this detailed examination of aerosol generation during several common medical procedures in a real-world healthcare setting, no detectable increase in particle generation was observed during mechanical ventilation, noninvasive ventilation, bronchoscopy, suctioning, or during the single NP swab collection and tracheostomy change. However, notable increases in particle mass concentration, number concentration, surface area deposition, and detectable particle levels were observed during NMA and sputum induction. A small increase in particle mass concentration (30% above baseline level) was observed during bronchoscopy, but no increases in particle number concentration or surface area deposition and no change in size distribution were observed. The lack of change in size distribution suggests routine use of respirators during these procedures may not be indicated. However, it should be noted that this study used particle size as a measure of aerosolization rather than an epidemiologic definition based on detected disease transmission.

High particle concentrations observed during both NMA and sputum induction are likely related to the use of a nebulizer during these procedures and probably represent nebulized saline or medications that escape the nebulizer during treatments,^33–36^ though coughing during sputum induction may also contribute to higher particle concentrations. Coughing also occurred during bronchoscopies. Although no viral or bacterial pathogens were recovered during the NMA and sputum induction procedures sampled for this study, the HCP administering these treatments were still exposed to aerosolized saline or medication.

Only one extubation, NP swab collection, and tracheostomy change were sampled. Capturing urgent or infrequent procedures is difficult in real-world studies, so we were fortunate to sample at least one of each procedure. Increases in particle number concentration and particle surface area deposition were observed for the single extubation, but sampling of additional procedures is needed to draw more firm conclusions about aerosol production.

None of the SKC BioSampler specimens collected during any of the sampled procedures were positive for a respiratory virus, even though 41% of the sampled procedures were performed on patients who had a recent clinical sample positive for a respiratory virus. Additionally, none of the BioSampler specimens were positive for MRSA, *C. difficile*, VRE, or another MDRO, even though 38% of the procedures were performed on patients who were on contact precautions for one or more of these organisms at the time of sampling; only clinically insignificant skin/environmental bacteria were identified. The failure to identify any significant viral or bacterial pathogens in any of the study specimens may indicate that few pathogens are present in medically generated aerosols. However, other potential explanations include: limited sensitivity of the SKC BioSamplers; the influence of ventilation/air handling characteristics of hospital rooms on particle behavior and flow patterns; and the fact that most patients were sampled several days after the onset of infection, after many had already received antimicrobials, which may have decreased viral shedding.

Strengths of this study include sampling multiple medical procedures during routine clinical care, use of multiple aerosol characterization instruments, and testing for both bacterial and viral pathogens. However, we also encountered several challenges and study limitations. First, small sample sizes for individual procedures precluded our ability to conduct statistical testing to allow us to rule out the influence of random sampling variation and draw conclusions about aerosol generation during procedures. Due to time and budget limitations, it was difficult to capture procedures that were performed infrequently (eg, tracheostomy change) or urgently (eg, intubation, extubation, NP swabs). Second, fewer than half of the study patients had a laboratory-proven infection, and most were sampled several days after the onset of infection, which may have limited capture of viable pathogens. Third, occasional instrument failure also resulted in data losses and incomplete samples. Fourth, although SKC BioSamplers are widely used for bioaerosol sampling and have superior rates of viral recovery compared to other methods of aerosol collection, such as filters and impactors,^37^ they have limited sampling efficiency for particles <1 µm or ≥9 µm in diameter.^38^ Although most bacterial particles would fall within this range, the ability of SKC BioSamplers to capture smaller viral particles may be limited.^37,39^ Finally, this work did not assess pathogen transmission, and it was conducted prior to the COVID-19 pandemic, so the findings do not assess SARS-CoV-2 transmission via aerosols.

In conclusion, the aerosol characterization data collected for this study do not show substantial increases in particle production or changes in particle size distribution during most procedures of interest, with the exception of NMA and sputum induction. In addition, no viral pathogens and only low-risk environmental bacterial pathogens were recovered from the air around patients during these procedures. These findings are similar to those of our earlier pilot study.^31^ Although it is difficult to make conclusions based on negative results, the findings of this study suggest that the risk of pathogen transmission due to small particle aerosol generation during these procedures may be lower than expected. Additional research is needed to replicate these results, especially for procedures with low numbers, and to examine the risk associated with other procedures that are commonly considered to be aerosol-generating.

## Supporting information

10.1017/ash.2026.10413.sm001 O’Neil et al. supplementary material 1 O’Neil et al. supplementary material

10.1017/ash.2026.10413.sm002 O’Neil et al. supplementary material 2 O’Neil et al. supplementary material

## Supplementary material

The supplementary material for this article can be found at https://doi.org/10.1017/ash.2026.10413.
