# Supplementary material for: Characterization of aerosols generated during suspected aerosol-generating procedures in healthcare settings
Source: Antimicrob Steward Healthc Epidemiol. 2026 May 21;6(1):e151. doi: 10.1017/ash.2026.10413 (PMC13199416; doi:10.1017/ash.2026.10413)
Supplement: O’Neil et al. supplementary material 1 — O’Neil et al. supplementary material [file S2732494X26104136sup001.docx]

**SUPPLEMENT TABLES**

**Supplement Table 1: Positive Bacterial culture results from among the 248 SKC BioSampler specimens.**

| Procedure | Baseline or Procedure | Bacterial organism  (Number of times detected) |
| --- | --- | --- |
| Extubation | Baseline | None detected |
|  | Procedure | None detected |
| Bronchoscopy | Baseline | *Micrococcus luteus^a^* (1) |
|  | Procedure | *Bacillus* species (2) |
|  |  | Gram positive cocci; not identifiable (2) |
|  |  | *Micrococcus luteus^a^* (1) |
|  |  | *Paenibacillus* species (1) |
| Bronchoscopy with | Baseline^b^ | Coagulase negative *Staphylococcus* (1) |
| Mechanical ventilation | Procedure | None detected |
| Mechanical ventilation | Procedure | *Bacillus* species (1) |
|  |  | *Micrococcus luteus* (1) |
|  |  | Coagulase negative *Staphylococcus* (2) |
| Noninvasive ventilation | Procedure | *Enterococcus faecalis* (1) |
|  |  | *Micrococcus luteus* (1) |
|  |  | Coagulase negative *Staphylococcus* (2) |
| Suctioning | Baseline | Gram positive cocci – type not identifiable (1) |
|  | Procedure | *Aerococcus viridans* (1) |
|  |  | *Lactobacillus* species (1) |
|  |  | *Micrococcus luteus* (1) |
|  |  | Coagulase negative *Staphylococcus* (2) |
| Nebulized medication | Baseline | *Bacillus* species (1) |
| administration |  | *Paenibacillus* species (1) |
|  |  | Coagulase negative *Staphylococcus* (1) |
|  | Procedure | *Kocuria kristinae* (1) |
|  |  | *Micrococcus luteus* (1) |
|  |  | Coagulase negative *Staphylococcus* (3) |
| Nebulized medication | Baseline^b^ | *Bacillus* species (1) |
| administration with |  | *Neisseria subflava* (1) |
| Noninvasive Ventilation |  | *Streptococcus* mitis (1) |
|  | Procedure | None detected |
| Sputum induction | Baseline | Coagulase negative *Staphylococcus* (1) |
|  | Procedure | *Bacillus* species (1) |
|  |  | Gram positive cocci – type not identifiable (2) |
|  |  | *Micrococcus luteus* (1) |
| Nasopharyngeal swab | Baseline | None detected |
| collection | Procedure | None detected |
| Tracheostomy change | Baseline | None detected |
|  | Procedure | Coagulase negative *Staphylococcus* (1) |

1. Samples were collected the same day in the same room, but were from two different patients/procedures, but were collected the same day in the same room.
2. Baseline sample for a planned procedure also counted as a procedure sample for a patient on MV or NIV.

**Supplement Table 2: Patient Demographic Characteristics and Comorbidities**

| Patient Characteristic | Patients  N=82 (%) |
| --- | --- |
| Age on date of sampling (years)  mean (SD) | 58.32 (13.8) |
| Comorbidities (any listed below) | 78 (95.1) |
| Asthma | 14 (17.1) |
| Chronic bronchitis | 1 (1.2) |
| COPD | 33 (40.2) |
| Congestive heart failure (CHF) | 22 (26.8) |
| Other cardiovascular disease | 28 (34.1) |
| Diabetes | 31 (37.8) |
| Kidney/renal disease | 27 (32.9) |
| Liver disease | 6 (7.3) |
| Cancer with treatment in last year^a^ | 9 (11.0) |
| HIV/AIDS | 2 (2.4) |
| History of transplant^b^ | 17 (20.7) |
| Systematic lupus erythematosus | 1 (1.2) |
| Rheumatoid arthritis | 3 (3.7) |

1. Includes non-melanoma skin cancer
2. Solid organ or BMT
