# Supplementary material for: Characterization of aerosols generated during suspected aerosol-generating procedures in healthcare settings
Source: Antimicrob Steward Healthc Epidemiol. 2026 May 21;6(1):e151. doi: 10.1017/ash.2026.10413 (PMC13199416; doi:10.1017/ash.2026.10413)
Supplement: O’Neil et al. supplementary material 2 — O’Neil et al. supplementary material [file S2732494X26104136sup002.docx]

**METHODS SUPPLEMENT**

**Aerosol Sampling Methods:**

All sampling equipment, including the P-Trak Ultrafine Particle Counter, SidePak AM520 Personal Aerosol Mobility Spectrometer, AeroTrak Portable Particle Counter, and the SKC BioSampler (mounted on a stand for stability), were loaded onto a specially-designed sampling cart (see Photo). The cart was designed with a small footprint, to reduce interference with the procedures being sampled. It was also designed to allow the sampler inlets to be lined up next to one another on the upper shelf of the cart, so that all would simultaneously collect air from the same area. During sampling, the cart was positioned approximately 3 feet away from the procedure with the sampler inlets facing the procedure area.


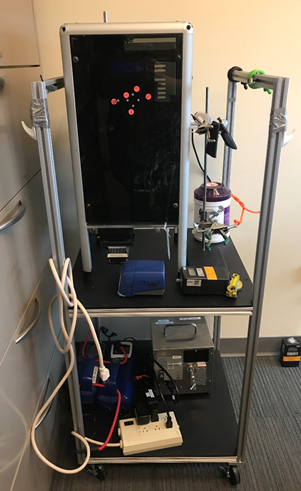


In aerosol science, particle mass, number, and concentration (size-resolved) provide complementary information, and the choice depends on the research objective. Mass concentration (e.g., PM₂.₅, PM₁₀) is most relevant for regulatory and health assessments, as it reflects bulk particulate exposure and is dominated by larger particles. In contrast, particle number concentration captures the abundance of particles, especially ultrafine particles (<100 nm), which contribute little to mass but are critical for understanding nucleation, combustion emissions, and health effects due to their high surface area and ability to penetrate deep into the lungs. Size-resolved concentration further links these metrics by describing how particles are distributed across diameters, providing insight into sources, formation mechanisms, and deposition behavior. Together, these measurements offer a comprehensive framework to characterize aerosol properties, processes, and impacts.

**Aerosol Sampling Instruments:**

- The TSI P-Trak Ultrafine Particle Counter 8525 is a condensation particle counter (CPC) that detects particles by enlarging them via alcohol vapor condensation and optical detection. It measures ultrafine particle number concentration over a size range of approximately 0.02–1 µm (20–1000 nm). The instrument actively samples air at a flow rate of ~0.1 L/min, with a typical time resolution of 1 second.
- The TSI SidePak AM520 Personal Aerosol Monitor is a laser photometer that estimates particle mass concentration (PM2.5) based on light scattering. It is sensitive primarily to particles in the ~0.1–10 µm range (calibrated to PM2.5 using a manufacturer or user-defined calibration factor). The SidePak operates with an internal pump at approximately 1.7 L/min, with user-selectable logging intervals (typically 1–60 seconds).
- The TSI AeroTrak Portable Particle Counter is an optical particle counter (OPC) that sizes particles based on light scattering intensity. It measures particles typically in discrete bins spanning approximately 0.3–10 µm(depending on configuration). In this study, particle counts were converted to an estimate of alveolar-deposited surface area (µm²/cm³) using established deposition models. The instrument samples at a flow rate of ~2.83 L/min (0.1 CFM), with time resolution typically 1 second.
- The TSI Aerodynamic Particle Sizer 3321 measures particle aerodynamic diameter using time-of-flight principles, covering a size range of 0.5–20 µm across multiple size bins. The APS actively samples air at a total flow of ~5 L/min (including sheath and sample flows), with a sample flow of ~1 L/min. Data were recorded at 1-second resolution.

**Influenza Culture Protocol:**

**Purpose:** to identify the presence of viable Influenza virus in BioSampler samples that had a positive BioFire Multiplex or GeneXpert Flu/RSV PCR test.

**Procedure:**

1. Culture medium preparation (medium was prepared daily during the sampling period to allow for real-time specimen testing)
   1. Seed 1 million MDCK (Madin-Darby Canine Kidney) cells in a 25cm^2^ culture flask.
   2. After 24 hours, wash the cells with phosphate buffered saline (PBS) to remove cell growth media
2. Specimen preparation
   1. Add 5.0 mL aliquot of the BioSampler collection fluid to the culture medium
   2. Spin the flask for 1 hour at 100xg
   3. Incubate for 1 hour at 37°C in a CO_2_ incubator.
3. Culture preparation
   1. Remove sample from the flask and wash the cells with PBS
   2. Add 5 ml of virus growth medium with TPCK trypsin
4. Incubation and assessment
   1. Incubate the specimen at 37°C for 7 days.
   2. Analyze for cell death or cytopathic effect at 24, 48, and 72 hours after inoculation and score from 0-4. CPE scores of ≥ 2 or more at 48 and 72 hours after inoculation were considered strong indicators of influenza virus replication in the specimen.
5. Detect influenza virus growth on day 7 by hemagglutination assay using turkey red blood cells
   1. Harvest the supernatant harvested and spin to remove cellular debris.
   2. Use the supernatant in a hemagglutination assay using 0.5% Turkey red blood cells (TRBC).
   3. Incubate two-fold serial dilutions of the supernatant in 50 microliters of PBS with 50 microliters of 0.5% TRBC for 30 minutes at room temperature. Blood cell agglutination will mean that influenza virus is present in the sample. The fold dilution at which the supernatant is positive for agglutination is the HA-titer and indicative of how much virus is present.
   4. Report influenza viral growth (yes/no). For samples with growth, report CPE scores for days 2 and 3 after inoculation and the HA-titer on day 7.
